# Advances in Nickel Nanoparticle Synthesis via Oleylamine Route

**DOI:** 10.3390/nano10040713

**Published:** 2020-04-09

**Authors:** Maria Heilmann, Hannes Kulla, Carsten Prinz, Ralf Bienert, Uwe Reinholz, Ana Guilherme Buzanich, Franziska Emmerling

**Affiliations:** 1 BAM Bundesanstalt für Materialforschung und -prüfung, Richard-Willstätter Strasse 11, D-12489 Berlin, Germany; 2 Humboldt-Universität zu Berlin, Department of Chemistry, Brook-Taylor Strasse 2, D-12489 Berlin, Germany

**Keywords:** nanoparticle synthesis, nickel nanoparticles, SAXS, TEM, XAS

## Abstract

Nickel nanoparticles are an active research area due to their multiple applications as catalysts in different processes. A variety of preparation techniques have been reported for the synthesis of these nanoparticles, including solvothermal, microwave-assisted, and emulsion techniques. The well-studied solvothermal oleylamine synthesis route comes with the drawback of needing standard air-free techniques and often space-consuming glassware. Here, we present a facile and straightforward synthesis method for size-controlled highly monodisperse nickel nanoparticles avoiding the use of, e.g., Schlenk techniques and space-consuming labware. The nanoparticles produced by this novel synthetic route were investigated using small-angle X-ray scattering, transmission electron microscopy, X-ray diffraction, and X-ray spectroscopy. The nanoparticles were in a size range of 4–16 nm, show high sphericity, no oxidation, and no agglomeration after synthesis.

## 1. Introduction

Nanoparticles are a class of functional materials with unique properties for a variety of chemical technologies and applications; thus, they are of great interest. The enhanced surface-area-to-volume ratio makes them excellent for use as catalysts, in analytical assays, and for antimicrobial applications [1,2,3]. In particular, metal nanoparticles like Au, Ag, Ru, Pt, Rh, Pd, and Ni nanoparticles are relevant in biomedical [4], antimicrobial [5], drug delivery [6], sensing/biosensing [7]. Moreover, metal nanoparticles are used in catalytic applications [6], including for the electrocatalytical hydrogen evolution reaction [8], acidic oxygen evolution [9], and the methanation (Sabatier) reaction [10].

During the Sabatier reaction, for example, energy surplus is converted to storable chemical energy via transformation of CO_2_ into CH_4_ [11]. Undesired CO_2_ and H_2_, generated using wind/solar energy, are exothermically converted to CH_4_ using a catalyst. Preferred catalyst materials include supported Rh, Ru, Ir, and Ni [10,12], with nickel being a promising candidate to replace the rare-earth metals as it is the most selective catalyst with a sufficiently high activity and a comparatively low price [13]. The advantage of nickel nanoparticles (NiNPs) on supporting materials compared to bulk nickel is the much higher surface-area-to-volume ratio, which offers a higher impact with less material and, therefore, less cost. NiNPs can also be used in different catalytic applications, such as hydrogenation of olefins [14], chemo-selective oxidative coupling of thiols [15], or alkaline hydrogen evolution reaction [16].

Size, shape, and the material polydispersity are crucial parameters for the application of NiNPs. Several synthesis routes have been developed, such as microwave-assistance [17], polyol processing [18], microemulsion [19], and solvothermal reduction [20]. To avoid the rapid oxidation of nickel to its oxides or hydroxides, an inert atmosphere during the synthesis is necessary. Solvothermal synthesis is a well-studied method for tuning the shape [21,22,23] and size of the nanoparticles. The influence of reaction parameters on the size of the nanoparticles has been intensively studied by changing the steric hindrance of the stabilizing agent [24], the chain length of the reducing agent amine [25], and by varying the amount of the reducing and stabilizing agent [20,26]. Donegan et al. showed that an increase in steric hindrance of the reducing agent (amine) leads to a decrease in nanoparticle size, and similar results were found by Park et al. for increasing steric hindrance of the stabilizing agent. Increasing the amount of stabilizing and reducing agents in a small range (e.g., stabilizing agent trioctylphosphine (TOP)/Ni ratios of 0.5–2 (LaGrow et al.) [20] or reducing agent oleylamine (OAm)/Ni ratios of 2–8 (Carenco et al.) [26]) resulted in a decrease in particle size. To understand the growth of NiNPs with different sizes in more detail, a comprehensive study with broader ranges of reagent ratios is therefore necessary.

Most syntheses described in the literature are conducted under standard air-free conditions using vacuum or inert gases like argon [22,24,27] or nitrogen [25,26,28] to remove potential air trapped in the solution and the gas phase, in space- and equipment-consuming three-necked flasks [22,25,26], or, alternatively, in an autoclave [21]. A simpler and much more space- and time-saving approach for synthesizing NiNPs under solvothermal conditions is needed that must still prevent the oxidation of nickel.

Here, we present a facile procedure for the solvothermal synthesis of NiNPs under near-air-free conditions, circumventing standard air-free conditions and space-consuming glassware. The procedure is conducted in glass vials with a septum and the reaction solution is purged with an N_2_ stream to remove trapped air in contrast to air-free Schlenk techniques. To investigate the influence on the size of the particles compared to synthetic routes reported in the literature, parameters like the amount of reducing agent and stabilizing agent, as well as concentration and reaction volume, were varied. We show that highly monodisperse non-oxidized NiNPs are achievable via this new facile procedure and that their size is tunable through the control of the reaction parameters.

## 2. Materials and Methods

### 2.1. Synthesis of Nickel Nanoparticles

**Materials**: Oleylamine (OAm, Acros, Geel, Belgium, C18-content 80–90%), trioctylphosphine (TOP, Sigma Aldrich, Karlsruhe, Germany, 90%), nickel(II) acetylacetonate (Ni(acac)_2_, Acros, Geel, Belgium, 96%), dibenzyl ether (DBE, Acros, Geel, Belgium, 99%), and n-hexane (Chemsolute, Renningen, Germany, 99%) were all used as received.

**Synthesis**: All reactions were carried out under a nitrogen atmosphere using a 20 mL glass vial sealed with a screw cap with a butyl/PTFE septum. These vials were heated using a dry block heater (IKA (Staufen, Germany), DB 5.6) on a heating plate (IKA (Staufen, Germany)). All syntheses were carried out at 5 or 10 mL volume to prevent the interference of any concentration or volume effects on the heating rate.

In a typical synthesis, 0.25 mmol Ni(acac)_2_ was added at room temperature to different volumes of OAm; the total volume was kept constant by adding DBE as necessary. After degassing the solution for 2 min by flushing it with a stream of N_2_, the solution was heated to 100 °C for 10 min. TOP/Ni ratios of 2–50 were added. This solution was degassed again using an N_2_ stream. Afterward, the solution was heated to 220 °C for 2 h. The mixture was cooled to room temperature. After the addition of excess acetone, the solution was centrifuged (4000 rpm, 4 min) and the supernatant was decanted. The black nanoparticle residue was then redispersed in n-hexane for analysis.

### 2.2. Analytical Methods

**SAXS analysis**: Small-angle X-ray scattering (SAXS) curves of the sample were collected on a Kratky-type SAXS instrument (SAXSess, Anton Paar, Graz, Austria), equipped with a sealed X-ray tube (λ_CuKα_ = 1.542 Å) and a microstrip X-ray detector (Mythen2 R detector system, Dectris, Baden-Daettwil, Switzerland). Samples were filled in a vacuum-tight glass capillary (diameter: 1 mm) and measured with an acquisition time of 10 min. The measured intensity was corrected for background contributions and the slit smearing effect using the software package SAXSquant4.2 (Anton Paar). The scattering vector q is defined in terms of the scattering angle 2θ and the wavelength λ of the incoming beam; thus, q = 4π/λ·sin θ. SAXS data analysis was performed with the SASfit program package 0.94.11 [29] using a model function that describes a homogeneous sphere with a Schulz–Zimm distribution of the radius, as shown in Equations (1)–(3).

(1) $$I\left(q,R\right)=f\left(R\right)P\left(q,R,\;\Delta \rho \right).$$

The scattering intensity I(q, R) in Equation (1) results from the form factor for a monodisperse sphere P(q, R, ∆ρ) in Equation (2) and the Schulz–Zimm distribution f(R) for the radius shown in Equation (3):

(2) $$P\left(q,R,\;\Delta \rho \right)={\left(\frac{4}{3}\pi R^{3}\Delta \rho^{3}\left(\frac{3\cdot (\sin\left(qR\right)-qR\;\cos\left(qR\right)}{{\left(qR\right)}^{3}}\right)\;\;\right)}^{2},$$

(3) $$f\left(R\right)=\frac{N}{R_{a}}{\left(\frac{R}{R_{a}}\right)}^{k-1}\frac{k^{k}e^{-kR/R_{a}}}{\Gamma \left(k\right)},$$

where R is the mean radius and k is based on the polydispersity, k = 1/σ^2^, with σ^2^ being the variance. R_a_ is the scaling parameter, defining the size distribution for large k values. N is the number of particles [30].

**TEM and STEM-EDS measurements**: Samples for transmission electron microscopy (TEM) and scanning transmission electron microscopy-X-ray spectroscopy (STEM-EDS) analyses were prepared by dropping the nickel nanoparticle solution on a lacey carbon-coated copper grid and allowing the solvent to evaporate. TEM and STEM-EDS analyses were performed on a Talos F200S microscope (Thermo Scientific, 200 kV, 1.4 Å point resolution).

**XRD measurements**: An acoustic levitator was used as a sample holder for the X-ray diffraction (XRD) measurements of colloidal nickel nanoparticle dispersions. The acoustic levitator was integrated in the μSpot beamline [31] at the BESSY II synchrotron (Helmholtz Centre Berlin for Materials and Energy, Berlin, Germany), as described elsewhere [32]. A monochromatized (Si 111) X-ray beam with a wavelength of 1.03358 Å and a beam size of 100 μm was used. The X-ray radiation was detected at a working distance of 200 mm with a 2D X-ray detector (MarMosaic, CCD 3072 × 3072 pixels). The diffraction images obtained were processed and converted into diagrams of scattered intensities versus the scattering vector q employing an algorithm from the FIT2D software [33]. q is defined by q = 4π/λ·sin θ with θ being the scattering half-angle and λ being the wavelength.

**XANES and EXAFS measurements**: X-ray near edge absorption spectroscopy (XANES) and extended X-ray absorption fine structure (EXAFS) investigations were also performed at the µSpot beamline at BESSY II. The samples were prepared by applying a colloidal nickel nanoparticle dispersion to boron nitride. The size of the resulting solid specimen was reduced with a mortar and pestle and the resulting specimen was pressed to a defined layer thickness of 1 mm. The determination of the absorption edge, the pre- and post-edge normalization of the absorption, the transformation of the experimental EXAFS data into k-space (wavenumber of the photoelectron), and the determination of the EXAFS oscillations χ(k) were performed with the program Athena [34]. The evaluation of the χ(k) function and the simulation of the scattering paths of the photoelectron were performed with the program Artemis.

## 3. Results

**Scheme 1:**
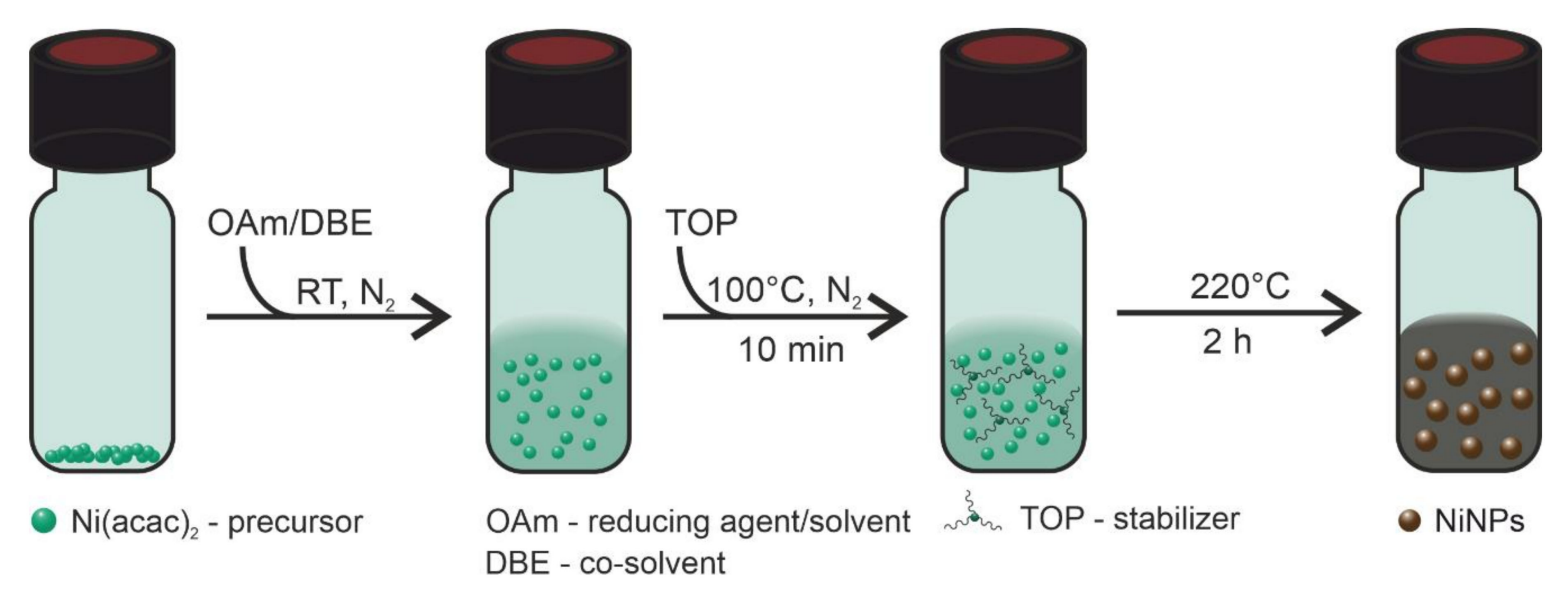
Schematic illustration of nickel nanoparticle (NiNP) synthesis conditions using nickel(II) acetylacetonate (Ni(acac)_2_) as precursor, Oam as reducing agent and solvent, dibenzyl ether (DBE) as co-solvent, and trioctylphosphine (TOP) as stabilizer, in 20 mL glass vials.

The typical synthesis procedure of the solvothermal reduction of Ni(acac)_2_ is schematically shown in Scheme 1. 

**Figure 1:**
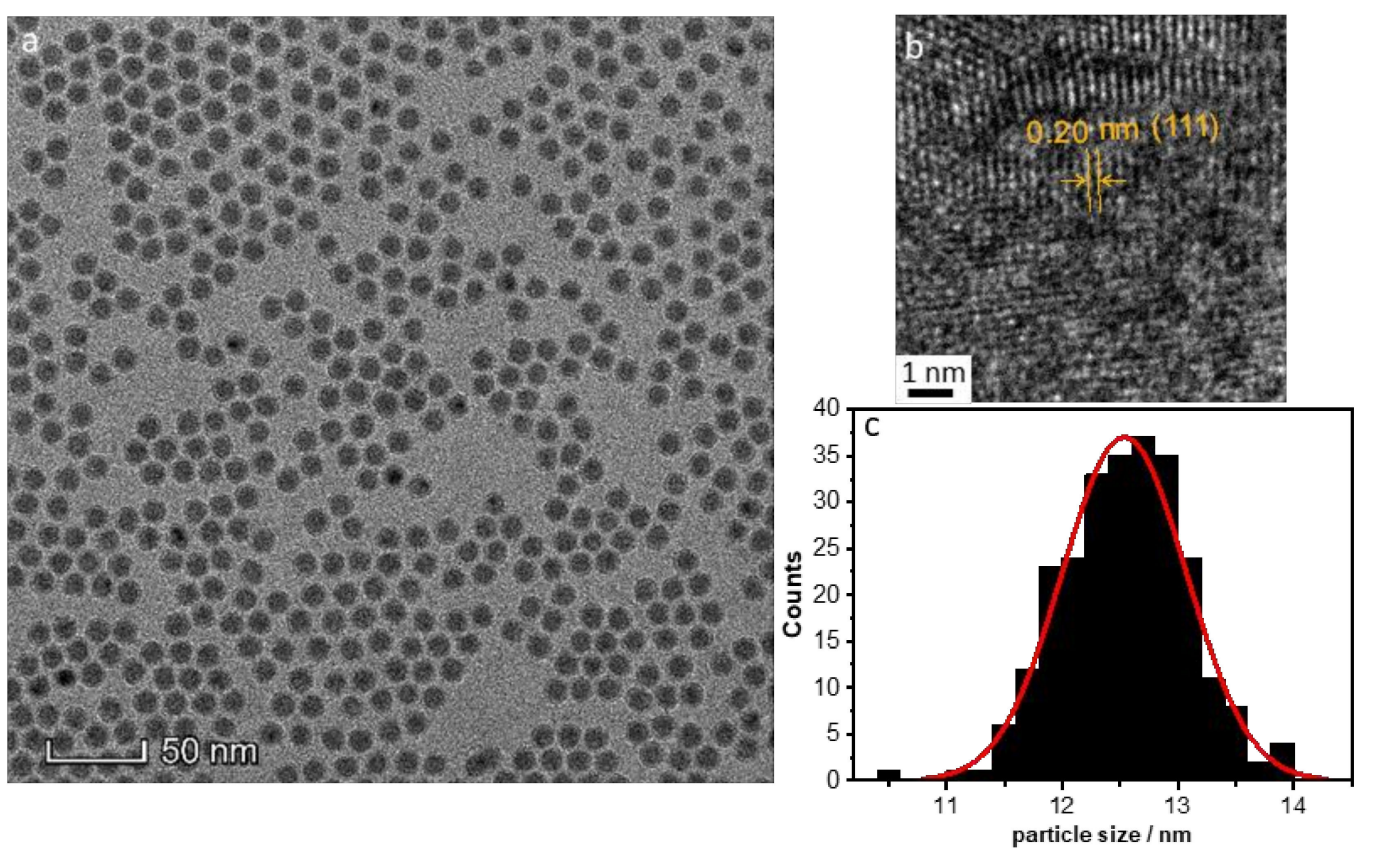
(**a**) TEM image of highly monodisperse NiNPs (diameter = 12.5 ± 0.5 nm); (**b**) zoomed-in TEM image revealing lattice fringes of 0.20 nm in NiNPs indicating fcc Ni (111); (**c**) particle size distribution of NiNPs.

This synthesis route enabled fine tuning of the sizes of NiNPs, such as in the case of the typically spherical 12.5 nm NiNPs shown in Figure 1a, obtained using the following conditions: 50 mmol/L Ni(acac)_2_, stabilizer TOP/Ni ratio of 2, OAm/Ni ratio of 10 as reducing agent and DBE as solvent, 220 °C, and a two-hour reaction time. The narrow size distribution (±0.5 nm) of the nanoparticles was confirmed by the TEM measurements of the nanoparticles, as shown in Figure 1c. Indication of the formation of the face-centered-cubic (fcc) nickel (111) phase was observed in the crystalline polydomain nanoparticles by means of the lattice fringes of 0.20 nm (Figure 1b).

**Figure 2:**
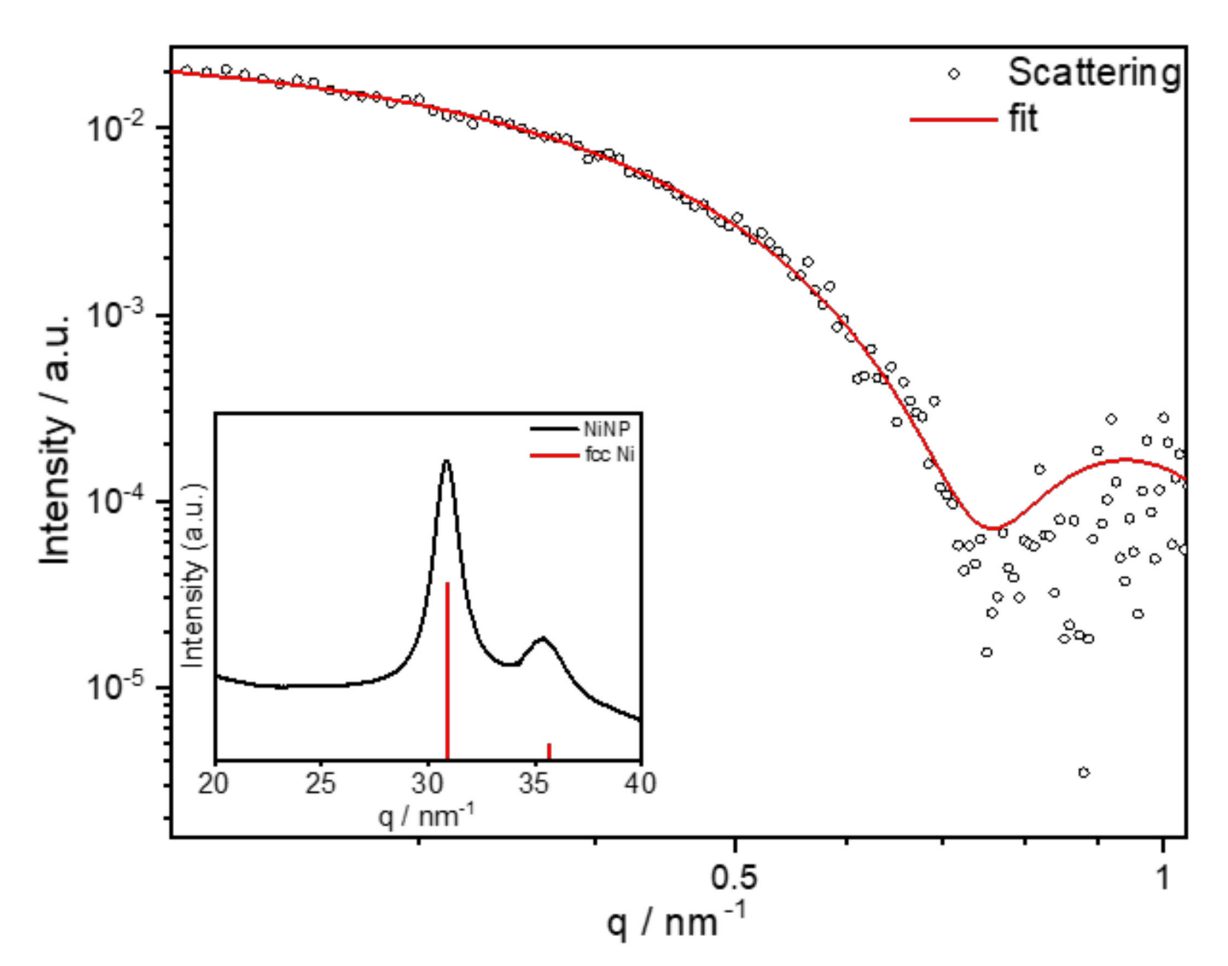
SAXS data of nickel nanoparticles dispersed in hexane with a curve fit according to a homogenous sphere with a Schulz–Zimm size distribution resulting in 11.8 nm, polydispersity 8%; (inset) XRD of NiNPs (black) as compared to the database entry JCPDS database PDF 04-0850 (red) for bulk nickel.

The analysis of the final nanoparticle dispersion using SAXS resulted in particle sizes of 11.8 nm with a polydispersity of 8%, using the Schulz–Zimm distribution (Figure 2). The XRD pattern of the synthesized nanoparticles is shown in Figure 2 (inset) in comparison to fcc Ni (JCPDS database, PDF 04-0850). The reflections at q = 44.5 and 51.8 nm^−1^ confirm the fcc structure of Ni. The breadth of the reflections is a result of the reduced crystallinity due to the relatively small crystallite size of the nanoparticles.

**Figure 3:**
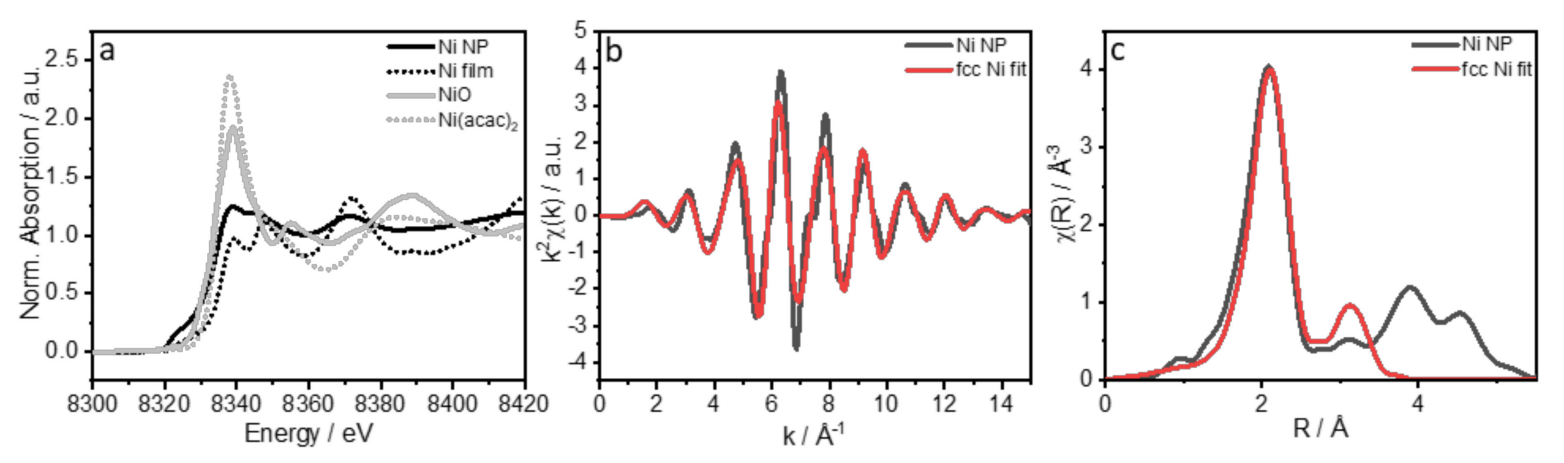
(**a**) Normalized Ni K-edge XANES-spectra of NiNPs (black straight), a Ni film (black dots), NiO (grey straight), and Ni(acac)_2_ used as precursor (grey dots); (**b**) visualization of k^2^-weighted extended X-ray absorption fine structure (EXAFS) oscillations in k-space of NiNPs (black) and fcc Ni bulk fit (red); (**c**) EXAFS oscillations as a function of the distance R obtained by Fourier transformation of NiNPs (black) and fcc Ni bulk fit (red).

To further investigate the local structure and the oxidation state of the nanoparticles, XANES and EXAFS measurements were conducted. Figure 3a shows the Ni K-edge XANES spectra of NiNPs compared to Ni(acac)_2_, NiO, and Ni standards. The similarity of the spectra of NiNPs and Ni film indicates the formation of pure Ni(0). The absence of an intense white line at around 8345 eV in the XANES spectrum of NiNPs and bulk Ni verifies the valence differences of nickel in the nanoparticles compared to the bivalent state in NiO and Ni(acac)_2_.This proves the formation of pure non-oxidized NiNPs [35,36].

EXAFS was carried out to gain an understanding of the local coordination geometry around nickel. In Figure 3b, the k^2^-weighted EXAFS spectra are compared to fcc Ni shown in k-space (k = wave number of the photoelectron). The spectra indicate that fcc Ni is formed in the crystalline domains in the NiNPs. Both the representation of the k-space and real space show that the experimentally obtained EXAFS data agree very well with the simulated structure of cubic surface-centered nickel (Figure 3c). There is a good correlation to fcc nickel for the coordination number, as well as for the interatomic distance, of the nickel atoms (Table 1). Taking these findings into account, one can conclude that the NiNPs consist of fcc Ni crystalline domains.

The ability to tune the size of the NiNPs is critical to ensure their suitability for large-scale industrial applications. We therefore explored various aspects of the synthesis method reported herein to optimize the conditions for tunability such as (i) time dependence of the particle formation, (ii) amount of reducing agent (OAm/Ni ratios of 5–122), (iii) amount of stabilizer (TOP/Ni ratio within 1–50), (iv) reaction volume dependence (5–20 mL) (see Scheme 1), and (v) long-term stability of the nanoparticles.

**Figure 4:**
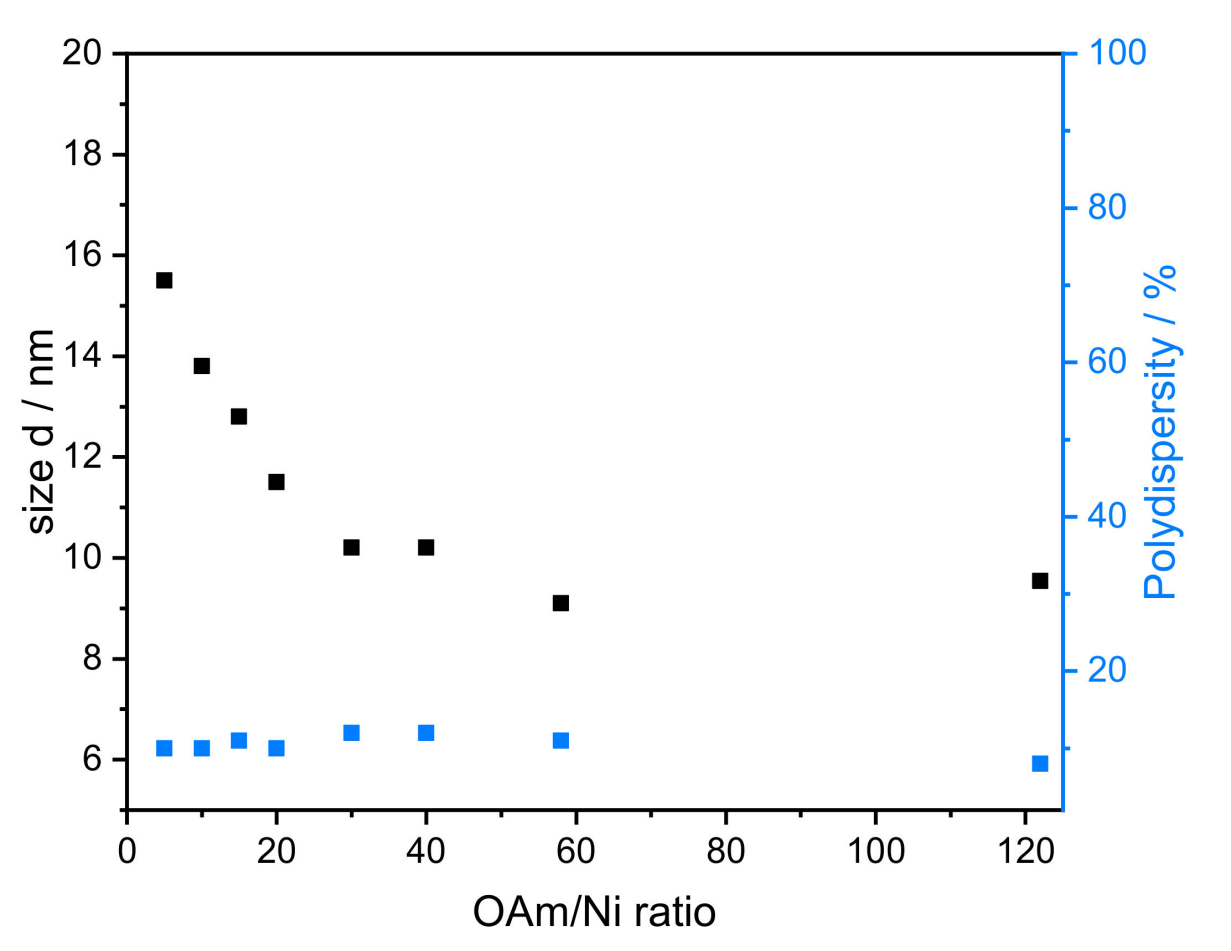
Nanoparticle size relative to OAm/Ni ratio with corresponding polydispersity, based on SAXS data. T = 220 °C, t = 2 h, [Ni] = 50 mmol/L, TOP/Ni = 1.5.

**Figure 5:**
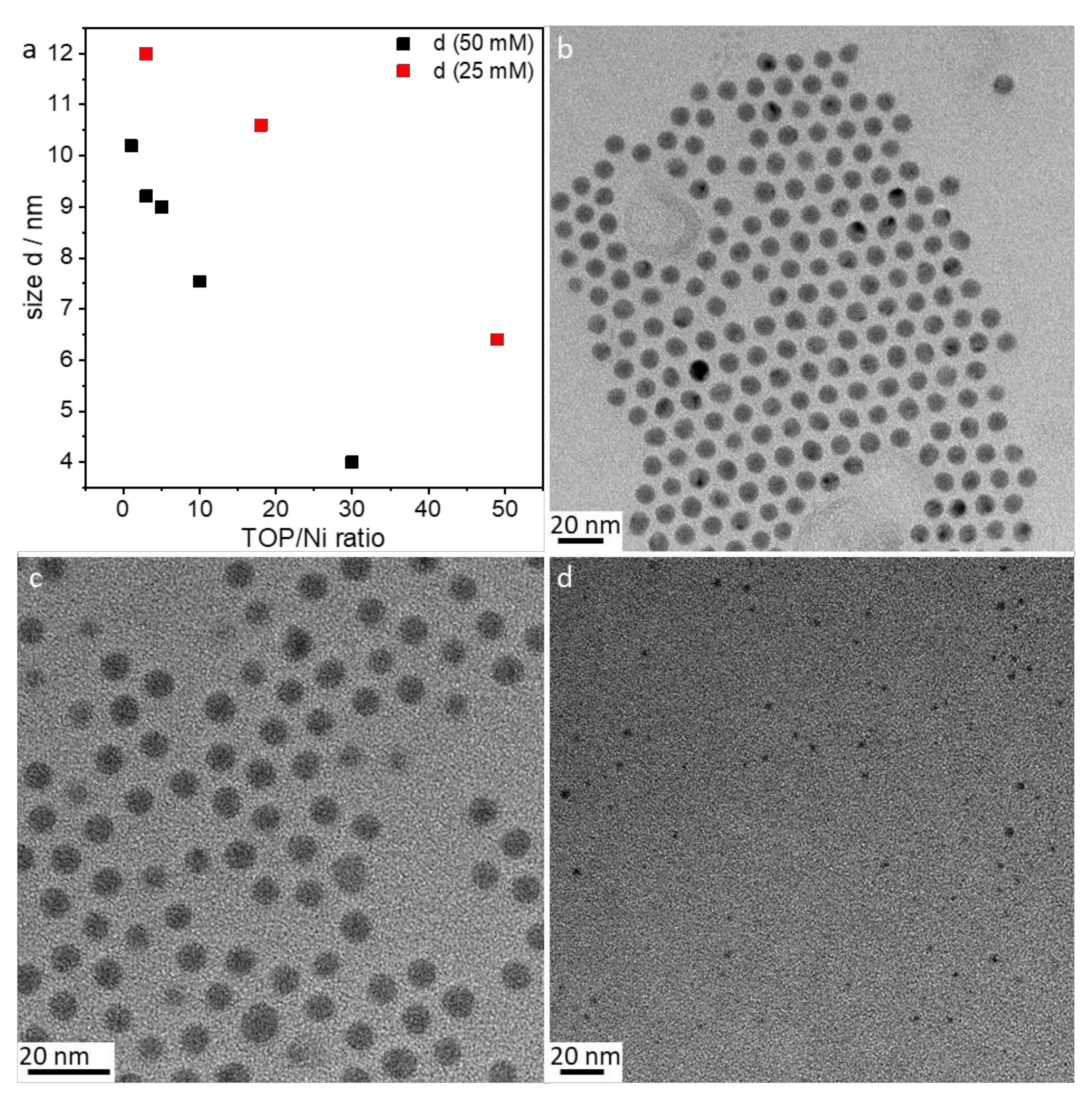
(**a**) Influence of TOP concentration on nanoparticle size: T = 220 °C, t = 2 h; (**b**–**d**) TEM images of NiNPs formed from 50 mmol/L Ni(acac)_2_ with TOP/Ni = 3 (**b**), TOP/Ni = 10 (**c**), and TOP/Ni = 30 (**d**).

(i) **Time dependence of particle formation**: For this purpose, the reaction using 50 mmol/L Ni(acac)_2_ in DBE with a reducing agent OAm/Ni ratio of 10 and stabilizer TOP/Ni ratio of 2 was heated to 220 °C and stopped after 30, 60, 90, and 120 min, precipitated, and redispersed in n-hexane. The particle sizes were determined using SAXS (see Table 2). The underlying SAXS-data are shown in Figure S1. SAXS data were used for the comparison as the dispersed nanoparticles were measured in solution, giving integrated information of the whole sample. The results indicate that the final size of the particles and the minimum size distribution had already been reached after one hour at 220 °C.

(ii) **Amount of reducing agent**: Dibenzyl ether (DBE) was considered a good additional solvent to OAm. It is non-toxic, stable at high temperature, and available in high purity. Based on SAXS data, the influence of the amount of OAm on the particle size can be studied using OAm/Ni ratios of 5–122 while keeping the TOP/Ni ratio at 1.5. The underlying SAXS-data are shown in Figure S2. The diameter of the particles varied between 8.0 and 15.5 nm (Figure 4), which correlates directly with the OAm amount. There was no significant change in the polydispersity when the OAm amount was varied. When no DBE was used, NiNPs with sizes of 10 and 8 nm were obtained for the highest concentrations of OAm (OAm/Ni ratios of 58 and 122), respectively.

(iii) **Amount of stabilizer**: The influence of the 1–50 TOP:Ni ratio was investigated using the following method based on SAXS data, which are shown in Figure S3. Two different concentrations (25 and 50 mmol/L) of Ni(acac)_2_ were tested. Increasing the TOP concentration resulted in both cases of decreasing particle sizes (Figure 5a). The decrease in particle size was steeper for the 50 mmol/L Ni(acac)_2_ concentration. TEM images revealed spherical particles narrowly distributed, well-separated, and arranged into two-dimensional networks due to the effective stabilization by TOP (Figure 5b–d). Upon increasing the TOP/Ni ratio to 30, smaller nanoparticles were formed in a significant amount, which were still well-separated but no longer formed networks.

(iv) **Reaction volume dependence**: Standard syntheses were conducted using a 5 mL solvent and 50 mmol/L nickel concentration. Triplicate experiments conducted within several months led to equally sized nanoparticles, as shown in Table 3. The underlying SAXS-data are shown in Figure S4. Increasing the reaction volume to 20 mL showed negligible effects on the nanoparticle size and size distribution.

**Figure 6:**
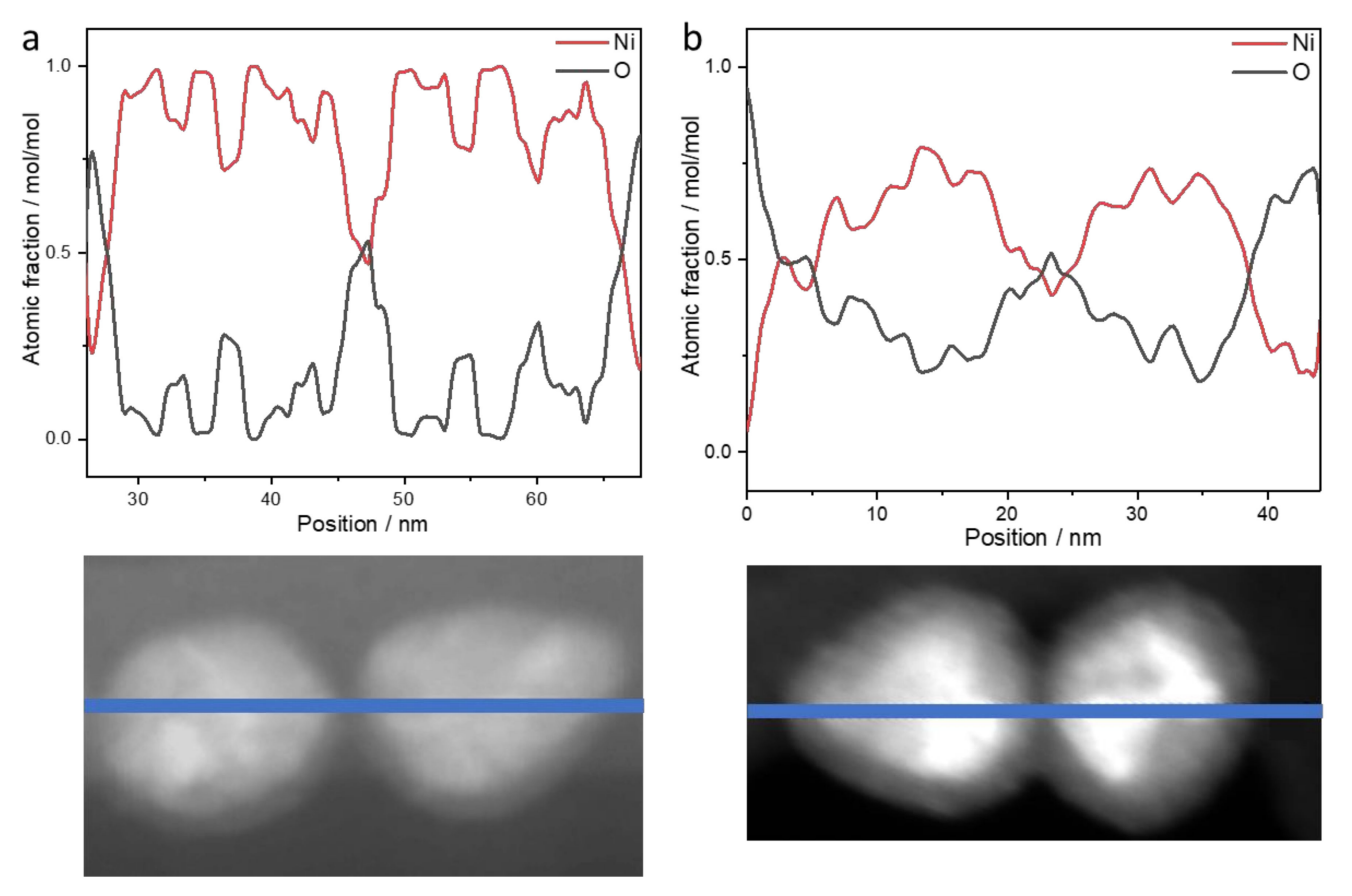
Element distribution of nickel and oxygen by EDS/STEM on the nanoparticles: (**a**) Two weeks after preparation; (**b**) stored in air for nine weeks after first measurement. The full STEM image and the regions of interest are shown in Figure S5.

**Figure 7:**
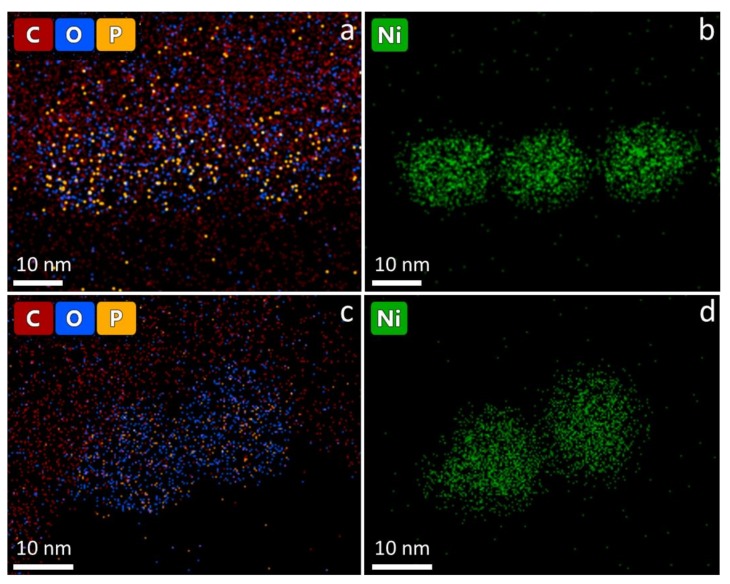
EDS mapping of NiNPs after synthesis: (**a**) Element distribution of carbon, oxygen, phosphorus, and (**b**) nickel. EDS mapping of NiNPs after nine weeks storage in air: (**c**) Element distribution of carbon, oxygen, phosphorus and (**d**) nickel.

(v) **Long-term stability of the nanoparticles**: NiNPs prepared with 50 mmol/L Ni(acac)_2_, TOP/Ni = 3, and OAm/Ni = 61 for 2 h at 220 °C were investigated using STEM and EDS. Figure 6 shows the distribution of elements (line scan indicated by blue line, K-lines) of nickel and oxygen on the nanoparticles two weeks after synthesis (Figure 6a) and after nine weeks of storage (Figure 6b) on the TEM grid. The expected distribution of nickel within the NiNPs is aligned with the contrast differences in the STEM image. Due to the lower local concentration after synthesis, the oxygen distribution around the nanoparticles demonstrates the ongoing oxidation process on the particle surface by showing higher intensities at the outer regions of the particles and less intensity at the inner regions of the particles. The overall atomic fraction of nickel in the particles decreases over time, indicated by a maximum amount of 100 mol/mol in the fresh nanoparticles in comparison to a maximum of 80 mol/mol in the altered particles. The carbon distribution is not analyzed in the line scan, as it shows strong intensities around the particles assignable to the lacey carbon film on the used copper TEM grid, but it is shown in the mapping image (Figure 7 left). The phosphorus distribution confirms the presence of stabilizer TOP on the particle surface leading to well-separated and organized nanoparticles.

## 4. Discussion

The synthesis conditions reported herein produced highly monodisperse 12.5 nm NiNPs with a size distribution of 0.5 nm (equivalent to a polydispersity of 4%), as presented in the TEM image in Figure 1. This is smaller than NiNPs synthesized in previous work [26], which were synthesized using the same amounts of TOP and OAm and gave 14 nm particles. Nanoparticles presented in that study in the size range of 9–13 nm have polydispersities of 7–10%, derived from TEM, which indicates that the use of the synthesis method in this work leads to smaller NiNPs with an improved size distribution.

Under typical reaction conditions, the final size of the nanoparticles was reached after 60 min at reaction temperature. However, Carenco et al. [26] showed in a TEM study that the surface of the particles was still very rough at this state. By extending the reaction time to two hours, the sphericity of the particles increased. This was attributed to an intra-particular reorganization of the nickel atoms in the outer layer (digestive ripening). A reaction time of two hours was chosen for our further investigations based on the findings of Ishizaki et al. [37]. As our synthesis was conducted using only an OAm/Ni ratio of 10 and DBE as solvent, the reaction time slightly increased, resulting in the formation of particles after 30 min. A slight ripening was observed over time, evident by the reduction in surface roughness. The shorter reaction time led to the formation of particles with smaller size but higher polydispersity.

Decreasing the amount of reducing agent OAm resulted in the formation of bigger particles with a comparable polydispersity. Compared to the synthesis using standard air-free methods, e.g., by Carenco et al. [26], smaller particles were observed at comparable amounts of reducing agent, which show higher sphericity at a comparable polydispersity.

There are two main reasons for the dramatic changes in size at lower OAm/Ni ratios (5–30). The first is a kinetic effect due to the nature of oleylamine acting as a reducing agent, so the amount present in the reaction directly correlates with the reaction rate. A reduced reaction rate by less reducing agent also decreases the nucleation rate and increases the probability of growth of the already-formed nickel clusters through aggregation. The most important role in these reaction kinetics is played by the amine group, which is responsible for the reduction process. This was demonstrated by Carenco et al. [28] via successful reduction with even shorter alkyl amines.

The second reason is the role of OAm as a stabilizer. The amine part interacts with the nickel surface while the hydrophobic alkyl chain provides steric hindrance [38]. A reduction in the amount of OAm increases the number of nucleation seeds in solution that are not sterically hindered from each other, which increases the chance of growth. However, when enough OAm is present (OAm/Ni = 30) in its role as stabilizer, increasing its amount only slightly changes the size of the particles from 10.2 to 8.0 nm. Only agglomerates were observed when an OAm/Ni ratio less than 5 was added. This is due to the fact that at least OAm/Ni = 3 is needed for a quantitative reduction of the nickel salt [39]. Larger particles (>21 nm) become ferromagnetic [40], which leads to magnetic agglomeration on the stirring bar. Using DBE as a solvent led to slightly bigger nanoparticles but showed no impact on the sphericity and size distribution.

Increasing the TOP concentration resulted in decreased particle sizes for both nickel concentrations (25 and 50 mmol/L). We hypothesize that due to the steric hindrance of TOP, the particles are more effectively stabilized, preventing them from continued growth through aggregation. The excess of OAm causes the nucleation to proceed so quickly that even a molar ratio of TOP/Ni equal to one is enough to stabilize the particles on the nickel surface. This leads to monodisperse nanoparticles with a polydispersity of only 10%. The decrease in particle size is steeper for the 50 mmol/L Ni(acac)_2_ concentration, which might be explained by noting the tendency of nuclei to coalesce grows with the number of growing seeds in the same volume. In contrast to former studies of LaGrow et al. [20], highly spherical nanoparticles with comparable sizes could be achieved. The region of strong effect of the stabilizing agent on the nanoparticle size is shifted to higher ratios (TOP/Ni = 3–50) compared to former studies of Carenco et al. [26]. In the latter study, the elevation in TOP/Ni ratio from 0.1 to 0.5 led to a strong change in particle size, whereas the size difference was negligible between TOP/Ni ratios of 0.5 and 5. Despite the strong effect of the reducing and stabilizing agent, the variation in sizes of the NiNPs is narrowly distributed within 4–16 nm, which is small compared to previous studies that produced nanoparticles within 9–32 nm, and the smaller size distribution reported in this paper indicates the stability of this improved synthesis method.

The size tunability of this facile synthesis route in addition to the reproducibility and high scalability is a significant step forward in the field of NiNPs. These advantages make this synthesis method interesting for industrial synthesis and catalytic applications. Furthermore, the long-term stability investigations showed oxidation occurring over time, suggesting that there are open sites on the nanoparticle surface accessible to the gas phase, which are prerequisites for good industrial catalysts. This confirms that these NiNPs have great potential to replace rare-earth metals in future catalyst materials. 

## 5. Conclusions

**Scheme 2:**
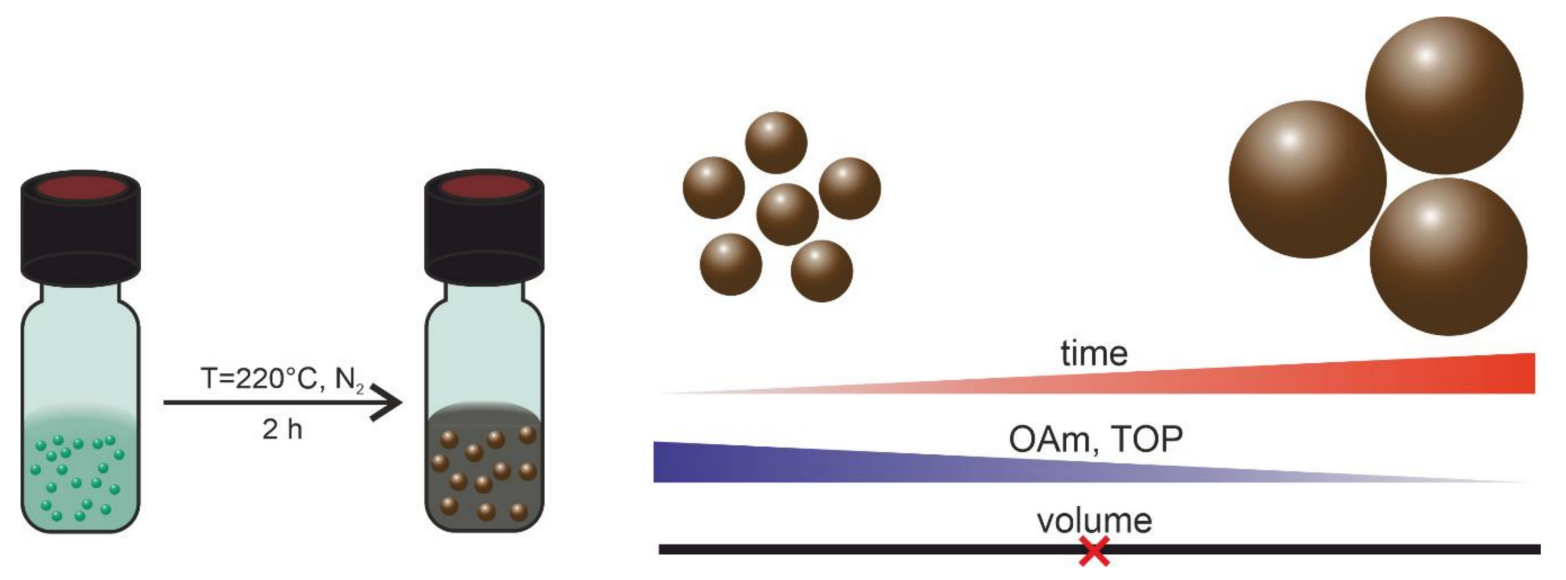
Schematic illustration of NiNP synthesis (left) and the impacts that (i) time, (ii) the amount of reducing agent OAm, (iii) the amount of stabilizer TOP, and (iv) the reaction volume have on the particle size (right).

Here we presented a facile synthesis method for size-controlled NiNPs, avoiding the use of Schlenk techniques and space-consuming labware. We investigated the formation of NiNPs using solvothermal reduction with different analytical techniques including SAXS, XRD, TEM, XANES, and EXAFS. We used an inert atmosphere over the solution in glass vials by degassing it for two minutes with a stream of nitrogen at room temperature and after ten minutes at 100 °C. With this facile and straightforward synthesis method, we could achieve highly monodisperse NiNPs in comparison to Ishizaki et al. [37] and smaller nanoparticles with higher sphericity compared to Carenco et al. [26]. The nanoparticles show only fcc Ni structure characteristics. Directly after synthesis, no oxidation of the particles was observed, eliminating the use of Schlenk techniques or the need to apply vacuum for the reaction. SAXS investigations were used to study the particle size by changing parameters like nickel salt concentration, amount of OAm and TOP, as well as total volume (see Scheme 2). In doing so we proved the size tunability of this new synthesis method, as well as the high scalability. The presented results confirm the applicability of this straightforward time-, equipment-, and space-saving method and will thus serve as the basis for the elucidation of the mechanisms of nanoparticle formation for future in situ experiments.

## Figures and Tables

**Table 1:** Comparison of the fitted EXAFS data of the NiNPs with the structural parameters of fcc Ni.

Coordination Number	Distance R/Å	
	NiNPs	Ni fcc
12	2.495	2.491
6	3.527	3.523
48	3.740	3.736

**Table 2:** Influence of reaction time on particle size and polydispersity.

Time/min	Size d/nm ^1^	Polydispersity/% ^1^
30	9.6	18
60	10.0	12
90	10.0	12
120	10.0	12

^1^ = SAXS data, [Ni] = 50 mmol/L.

**Table 3:** Volume-independent nanoparticle size. [Ni] 50 mmol/L.

Volume/mL	Size d/nm	Polydispersity/%
5	9.2	8
5	9.4	8
5	8.8	11
10	9.4	9
20	9.6	11

## Supplementary Materials

The following are available online at https://www.mdpi.com/2079-4991/10/4/713/s1, Figure S1: SAXS data of particles after different reaction times with corresponding Schulz–Zimm-fit (red lines), Figure S2: Influence of the OAm:Ni ratio on the particle size of nickel nanoparticles. T = 220 °C, t = 2 h, [Ni] = 50 mmol/L, TOP = 1.5 eq. SAXS data with corresponding Schulz–Zimm fit (red lines), Figure S3: SAXS data with corresponding Schulz–Zimm fit (red lines). Influence of TOP:Ni ratio on the nanoparticle size. T= 220 °C, t = 2 h, [Ni] = 50 mmol/L (left) and [Ni] = 25 mmol/L (right), pure OAm, Figure S4: SAXS data with corresponding Schulz–Zimm fit (red lines). Volume-independent nanoparticle size. T = 220 °C, t = 2 h, [Ni] = 50 mmol/L, TOP = 1.5 eq, pure OAm, Figure S5: STEM images of NiNPs after synthesis (left) and after nine weeks of storage in air (right), and selected regions (white rectangle) used for EDS measurements.

## References

[1] Kawi S., Kathiraser Y., Ni J., Oemar U., Li Z.W., Saw E.T. (2015). Progress in Synthesis of Highly Active and Stable Nickel-Based Catalysts for Carbon Dioxide Reforming of Methane. ChemSusChem.

[2] Bedwell T.S., Whitcombe M.J. (2016). Analytical applications of MIPs in diagnostic assays: Future perspectives. Anal. Bioanal. Chem..

[3] Deshmukh S.P., Patil S.M., Mullani S.B., Delekar S.D. (2019). Silver nanoparticles as an effective disinfectant: A review. Mater. Sci. Eng. C Mater. Biol. Appl..

[4] Kalimuthu K., Cha B.S., Kim S., Park K.S. (2020). Eco-friendly synthesis and biomedical applications of gold nanoparticles: A review. Microchem. J..

[5] Rai M., Yadav A., Gade A. (2009). Silver nanoparticles as a new generation of antimicrobials. Biotechnol. Adv..

[6] Naseem K., Begum R., Farooqi Z.H. (2018). Platinum nanoparticles fabricated multiresponsive microgel composites: Synthesis, characterization, and applications. Polym. Compos..

[7] Yu L., Li N. (2019). Noble Metal Nanoparticles-Based Colorimetric Biosensor for Visual Quantification: A Mini Review. Chemosensors.

[8] Bae S.Y., Mahmood J., Jeon I.Y., Baek J.B. (2020). Recent advances in ruthenium-based electrocatalysts for the hydrogen evolution reaction. Nanoscale Horiz..

[9] Guo H.Y., Fang Z.W., Li H., Fernandez D., Henkelman G., Humphrey S.M., Yu G.H. (2019). Rational Design of Rhodium-Iridium Alloy Nanoparticles as Highly Active Catalysts for Acidic Oxygen Evolution. ACS Nano.

[10] Frontera P., Macario A., Ferraro M., Antonucci P. (2017). Supported Catalysts for CO2 Methanation: A Review. Catalysts.

[11] Maroufmashat A., Fowler M. (2017). Transition of Future Energy System Infrastructure; through Power-to-Gas Pathways. Energies.

[12] Rönsch S., Schneider J., Matthischke S., Schlüter M., Götz M., Lefebvre J., Prabhakaran P., Bajohr S. (2016). Review on methanation—From fundamentals to current projects. Fuel.

[13] Mills G.A., Steffgen F.W. (1974). Catalytic Methanation. Catal. Rev..

[14] Dhakshinamoorthy A., Pitchumani K. (2008). Clay entrapped nickel nanoparticles as efficient and recyclable catalysts for hydrogenation of olefins. Tetrahedron Lett..

[15] Saxena A., Kumar A., Mozumdar S. (2007). Ni-nanoparticles: An efficient green catalyst for chemo-selective oxidative coupling of thiols. J. Mol. Catal. Chem..

[16] Gong M., Wang D.-Y., Chen C.-C., Hwang B.-J., Dai H. (2016). A mini review on nickel-based electrocatalysts for alkaline hydrogen evolution reaction. Nano Res..

[17] Eluri R., Paul B. (2012). Microwave assisted greener synthesis of nickel nanoparticles using sodium hypophosphite. Mat. Lett..

[18] Logutenko O.A., Titkov A.I., Vorob’ev A.M., Shundrina I.K., Yukhin Y.M., Lyakhov N.Z. (2018). Synthesis of Nickel Nanoparticles by the Reduction of Its Salts Using the Modified Polyol Method in the Presence of Sodium Polyacrylates with Various Molecular Weights. Russ. J. Gen. Chem..

[19] Chen D.-H., Wu S.-H. (2000). Synthesis of nickel nanoparticles in water-in-oil microemulsions. Chem. Mater..

[20] LaGrow A.P., Ingham B., Toney M.F., Tilley R.D. (2013). Effect of Surfactant Concentration and Aggregation on the Growth Kinetics of Nickel Nanoparticles. J. Phys. Chem. C.

[21] Liu S., Mei J., Zhang C., Zhang J., Shi R. (2018). Synthesis and magnetic properties of shuriken-like nickel nanoparticles. J. Mater. Sci. Technol..

[22] Wang Z.C., Chen Y.Z., Zeng D.Q., Zhang Q.F., Peng D.L. (2016). Solution synthesis of triangular and hexagonal nickel nanosheets with the aid of tungsten hexacarbonyl. CrystEngComm.

[23] LaGrow A.P., Ingham B., Cheong S., Williams G.V.M., Dotzler C., Toney M.F., Jefferson D.A., Corbos E.C., Bishop P.T., Cookson J. (2012). Synthesis, Alignment, and Magnetic Properties of Monodisperse Nickel Nanocubes. J. Am. Chem. Soc..

[24] Park J., Kang E., Son S.U., Park H.M., Lee M.K., Kim J., Kim K.W., Noh H.J., Park J.H., Bae C.J. (2005). Monodisperse Nanoparticles of Ni and NiO: Synthesis, Characterization, Self-Assembled Superlattices, and Catalytic Applications in the Suzuki Coupling Reaction. Adv. Mat..

[25] Donegan K.P., Godsell J.F., Otway D.J., Morris M.A., Roy S., Holmes J.D. (2012). Size-tuneable synthesis of nickel nanoparticles. J. Nanopart. Res..

[26] Carenco S., Boissiére C.D., Nicole L., Sanchez C.M., Le Floch P., Mézailles N. (2010). Controlled Design of Size-Tunable Monodisperse Nickel Nanoparticles. Chem. Mater..

[27] Chen G., Desinan S., Rosei R., Rosei F., Ma D. (2012). Synthesis of Ni-Ru Alloy Nanoparticles and Their High Catalytic Activity in Dehydrogenation of Ammonia Borane. Chem. Eur. J..

[28] Carenco S., Labouille S., Bouchonnet S., Boissiere C., Le Goff X.F., Sanchez C., Mezailles N. (2012). Revisiting the Molecular Roots of a Ubiquitously Successful Synthesis: Nickel(0) Nanoparticles by Reduction of [Ni(acetylacetonate)(2)]. Chem. Eur. J..

[29] Breßler I., Kohlbrecher J., Thünemann A.F. (2015). SASfit: A tool for small-angle scattering data analysis using a library of analytical expressions. J. Appl. Crystallogr..

[30] Kohlbrecher J. (2018). SASfit: A Program. for Fitting Simple Structural Models to Small Angle Scattering Data.

[31] Paris O., Li C., Siegel S., Weseloh G., Emmerling F., Riesemeier H., Erko A., Fratzl P. (2007). A new experimental station for simultaneous X-ray microbeam scanning for small-and wide-angle scattering and fluorescence at BESSY II. J. Appl. Crystallogr..

[32] Wolf S.E., Leiterer J., Kappl M., Emmerling F., Tremel W. (2008). Early homogenous amorphous precursor stages of calcium carbonate and subsequent crystal growth in levitated droplets. J. Am. Chem. Soc..

[33] Hammersley A., Brown K., Burmeister W., Claustre L., Gonzalez A., McSweeney S., Mitchell E., Moy J.-P., Svensson S., Thompson A. (1997). Calibration and application of an X-ray image intensifier/charge-coupled device detector for monochromatic macromolecular crystallography. J. Synchrotron Radiat..

[34] Ravel B., Newville M. (2005). ATHENA, ARTEMIS, HEPHAESTUS: Data analysis for X-ray absorption spectroscopy using IFEFFIT. J. Synchrotron Radiat..

[35] Sakamoto T., Kishi H., Yamaguchi S., Matsumura D., Tamura K., Hori A., Horiuchi Y., Serov A., Artyushkova K., Atanassov P. (2016). Mechanism Study of Hydrazine Electrooxidation Reaction on Nickel Oxide Surface in Alkaline Electrolyte by In Situ XAFS. J. Electrochem. Soc..

[36] Winnischofer H., Rocha T.C., Nunes W.C., Socolovsky L.M., Knobel M., Zanchet D. (2008). Chemical synthesis and structural characterization of highly disordered Ni colloidal nanoparticles. ACS Nano.

[37] Ishizaki T., Yatsugi K., Akedo K. (2016). Effect of Particle Size on the Magnetic Properties of Ni Nanoparticles Synthesized with Trioctylphosphine as the Capping Agent. Nanomaterials.

[38] Mourdikoudis S., Liz-Marzán L.M. (2013). Oleylamine in Nanoparticle Synthesis. Chem. Mater..

[39] Cheng G., Puntes V.F., Guo T. (2006). Synthesis and self-assembled ring structures of Ni nanocrystals. J. Colloid Interf. Sci..

[40] Zhang H., Ding J., Chow G., Ran M., Yi J. (2009). Engineering magnetic properties of Ni nanoparticles by non-magnetic cores. Chem. Mater..

