# Supplementary material for: Advances in Nickel Nanoparticle Synthesis via Oleylamine Route
Source: Nanomaterials (Basel). 2020 Apr 9;10(4):713. doi: 10.3390/nano10040713 (PMC7221568; doi:10.3390/nano10040713)
Supplement: Supplementary file 1 [file nanomaterials-10-00713-s001.pdf]

nanomaterials

Supplementary Materials

Advances in Nickel Nanoparticle Synthesis via Oleylamine Route

Maria Heilmann [1,2], Hannes Kulla [1], Carsten Prinz [1], Ralf Bienert [1], Uwe Reinholz [1], Ana Guilherme Buzanich [1] and Franziska Emmerling [1,2,*]

[1] BAM Bundesanstalt für Materialforschung und -prüfung, Richard-Willstätter Strasse 11, D-12489 Berlin, Germany; maria.heilmann@bam.de (M.H.); hkulla11@gmail.com (H.K.); Carsten.Prinz@bam.de (C.P.); Ralf.Bienert@bam.de (R.B.); Uwe.Reinholz@bam.de (U.R.); Ana.Buzanich@bam.de (A.G.B.)

[2] Humboldt-Universität zu Berlin, Department of Chemistry, Brook-Taylor Strasse 2, D-12489 Berlin, Germany

* Correspondence: franziska.emmerling@bam.de

(i) SAXS data of nickel nanoparticles (NPs) at different reaction times corresponding to the sizes and distributions in Table 2.

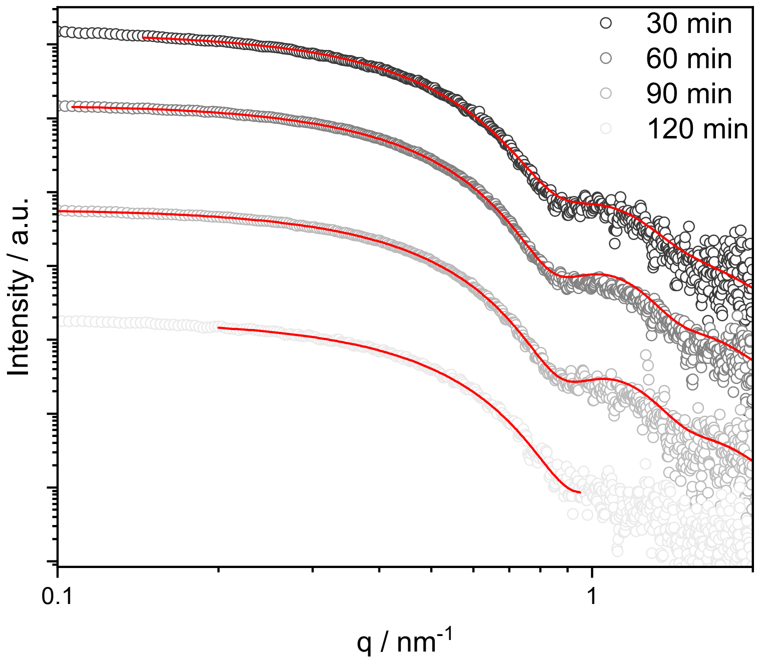

Figure S1. SAXS data of particles after different reaction times with corresponding Schulz–Zimm fit (red lines).

(ii) SAXS data of Ni NPs synthesized using different amounts of oleylamine (OAm) corresponding to Figure 4.

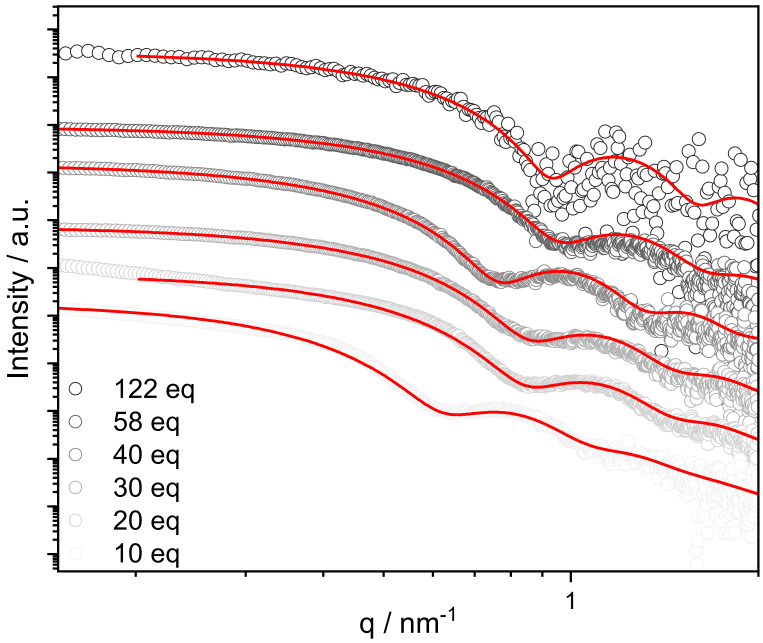

Figure S2. Influence of the OAm:Ni ratio on the particle size of nickel nanoparticles. T = 220 °C, t = 2 h, [Ni] = 50 mmol/L, TOP = 1.5 eq. SAXS data with corresponding Schulz–Zimm fit (red lines).

(iii) SAXS data of Ni NPs synthesized using different amounts of stabilizer trioctylphosphine (TOP) at two different Ni concentrations corresponding to Figure 5.

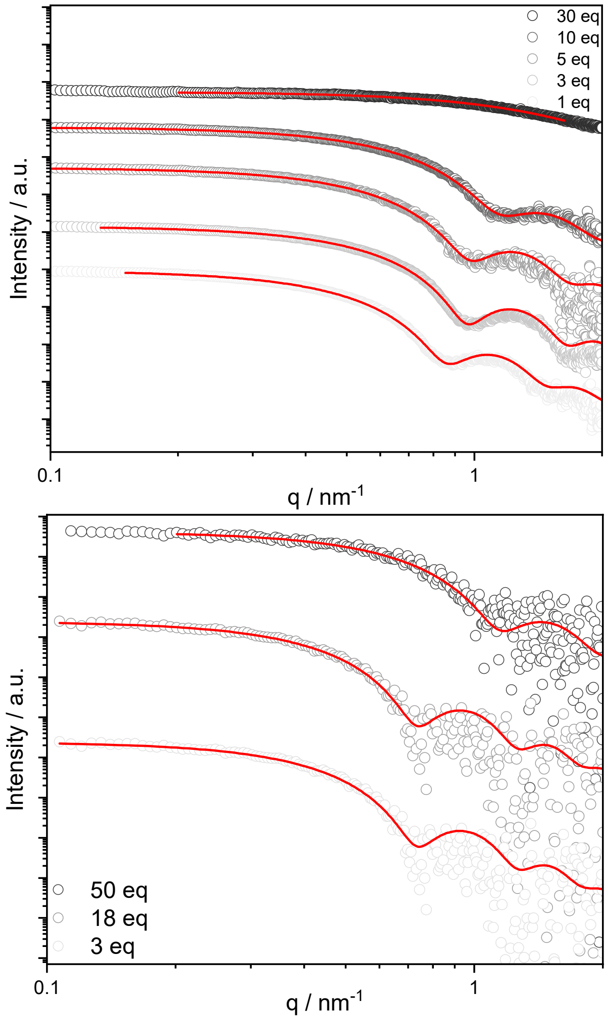

Figure S3. SAXS data with corresponding Schulz–Zimm fit (red lines). Influence of TOP:Ni ratio on the nanoparticle size. T= 220 °C, t = 2 h, [Ni] = 50 mmol/L (left) and [Ni] = 25 mmol/L (right), pure Oam.

(iv) SAXS data of Ni NPs corresponding to Table 3 synthesized using different reaction volumes.

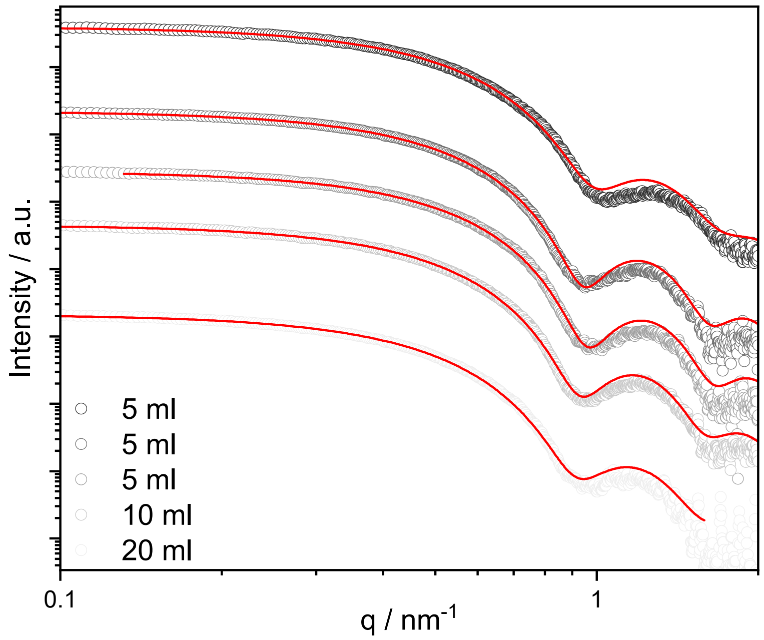

Figure S4. SAXS data with corresponding Schulz–Zimm fit (red lines). Volume-independent nanoparticle size. T = 220 °C, t = 2 h, [Ni] = 50 mmol/L, TOP = 1.5 eq, pure Oam.

(v) STEM data of Ni NPs taken directly after synthesis and after nine weeks of storage corresponding to Figure 6 and 7:

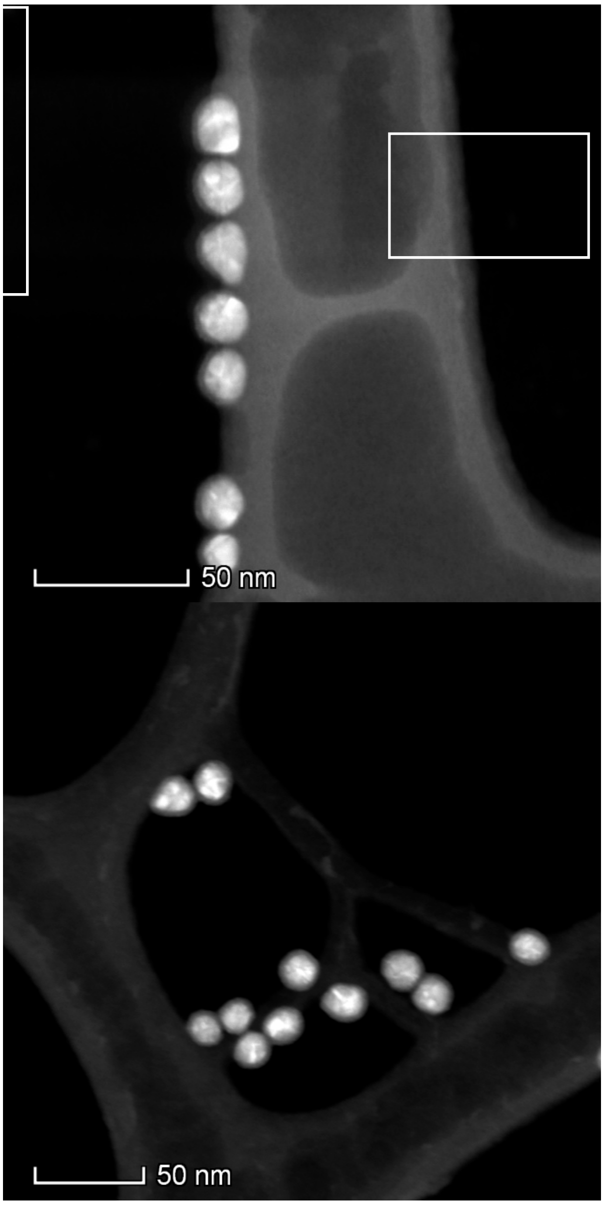

Figure S5. STEM images of Ni nanoparticles after synthesis (left) and after nine weeks of storage in air (right), and selected regions (white rectangle) used for EDS measurements.
